# Gamma irradiation effects on in vitro shoot cultures of *Dianthus caryophyllus* L. and molecular characterization of mutants using ISSR markers

**DOI:** 10.1038/s41598-026-45205-w

**Published:** 2026-04-06

**Authors:** Puja Sharma, Sita Ram Dhiman, Youdh Chand Gupta, Priyanka Sharma, Rajinder Kaur, Shabnam Pangtu, Poonam Sharma

**Affiliations:** 1 Department of Floriculture and Landscaping, College of Horticulture, Dr. Yashwant Singh Parmar University of Horticulture and Forestry, Nauni, Solan, Himachal Pradesh 173230 India; 2 Department of Floriculture and Landscaping, Rani Lakshmi Bai Central Agricultural University, Jhansi, Uttar Pradesh 284003 India; 3 Department of Biotechnology, Dr. Yashwant Singh Parmar University of Horticulture and Forestry, Nauni, Solan, Himachal Pradesh 173230 India

**Keywords:** Carnation, Gamma rays, ISSR, Molecular markers, Mutants, Variation, Biological techniques, Biotechnology, Genetics, Molecular biology, Plant sciences

## Abstract

The current investigation was carried out to study the effect of different doses of gamma irradiation on *in vitro* shoot cultures of carnation (*Dianthus caryophyllus* L.) and characterization of mutants using ISSR markers. The trial was undertaken at the experimental farm and laboratory of the Department of Floriculture and Landscaping, Dr. YS Parmar University of Horticulture and Forestry, Nauni, Solan (HP). The experiment on ten carnation genotypes i.e. 7 mutants, 3 parents viz.; ‘Dark Rendezvous’, ‘Madras’ and ‘Tempo’ was laid out in Randomized Block Design (RBD) under polyhouse conditions. All the mutants developed after the irradiation with gamma rays showed variation in color. In this study, different morphological parameters of the mutants and their parents were recorded. Among different mutants, maximum plant height was recorded in mutant of ‘Dark Rendezvous’ i.e. ‘Rendez-Vous A_2_’ (67.70 cm) and maximum flower size in ‘Rendez-Vous C_2_’ (8.40 cm). Duration of flowering was recorded maximum in ‘Tempo A_1_’ (67.67 days) and vase life in ‘Rendez-Vous C_1_’ (14.79 days). For the majority of the characters analyzed, all mutants performed better in comparison to parents. Moreover, for the characterization of mutants at the molecular level, ten ISSR primers were used and DNA polymorphism was observed with four primers. A total of fifteen polymorphic bands were produced by these four primers. No monomorphic band was observed with any primer resulting in 100% polymorphism. Variations in the plant material can be explained through genetic polymorphism. These results directly strengthen the significance of mutation breeding in developing improved carnation cultivars and substantiate the use of molecular characterization for validating genetic variation. Furthermore, they emphasize the scope for the commercial expansion of carnation cultivation through the selection of promising mutants.

## Introduction

Carnation (*Dianthus caryophyllus* L.) is one of the most widely grown floricultural crops throughout the world. This plant is a half-hardy, herbaceous perennial distinguished by branching stems and a perpetual flowering habit. In temperate regions, this crop can be grown all year round, making it highly suitable for cut flower production. Colombia, Kenya and China are the main carnation-producing countries in the world. In India, carnations are grown in the Nilgiris and Kodaikanal regions of Tamil Nadu and in the Solan, Sirmour, Shimla, Bilaspur and Chamba districts of Himachal Pradesh^1^. Carnation is widely preferred by commercial growers due to its extended vase life and suitability for long-distance markets. Considering the rising global market demand for cut flowers, there exists a need to develop novel cultivars. Conventional breeding has been employed for the development of new cultivars of commercial significance. However, it has barriers, as it is labour-intensive and more time-consuming^2^. Mutation breeding is one of the most important approaches for developing new cultivars in carnation. Mutations in carnations are often induced by the use of ionising radiation, such as gamma rays and X-rays^3–6^. Stable mutants in carnation cultivars have been produced by splitting cuttings lengthwise into two, then irradiating them, and culturing in a medium.

Induction of mutations combined with plant tissue culture approaches provides a significant advantage in crops like carnation, which are vegetatively propagated. *In vitro* culture of irradiated explants has been used to increase the novelty of colours in carnation. Simard et al.^7^ utilized organogenesis to develop variants of carnation from irradiated petals. A dose of 40 Gy was reported to produce variants of horticultural interest on the fourth day of culture in the cultivar ‘Niky’^8^. Similarly, after *in vitro* X-ray irradiation of nodes, Cassells et al.^9^ found that the carnation cv. ‘Mystere’ produced useful flower colour mutations. Additionally, they identified vigorous and stable mutants using *in vitro* subculturing. Internodal segments of the ‘Mini Pinky’ carnation cultivar were exposed to gamma and X-ray radiation (5–30 Gy) and then grown on regeneration media by Jerzy and Zalewska^10^.They observed that the number of adventitious shoots that emerged from irradiated explants ranged from 0 to 5, while most of the internodal segments only produced one adventitious shoot. *In vitro* shootlets of carnation were irradiated with doses of 20, 40, 60 and 80 Gy of gamma radiation. Leaves were excised from irradiated shootlets and used as explants for further culturing. The percentage of survival decreased when the gamma radiation dose was raised, and the 40Gy treatment was found to be most effective for mutagenesis^11^.

Conventionally, morphological markers have been utilized for differentiation and characterization of varieties in horticultural crops. However, molecular markers have several advantages compared to the morphological markers due to their higher polymorphism and environmental stability^12^. Molecular markers, especially ISSR (Inter Simple Sequence Repeats) provide significant information associated with population genetics and their evolution. ISSRs are DNA segments bounded by microsatellite sequences from both ends and exhibit high informativeness, polymorphism and reproducibility^2^. Molecular markers are widely used in plant breeding for genetic diversity assessment, varietal identification, gene mapping and identification of phylogenetic relationships among germplasm^13^. Recently, there has been increasing emphasis on the identification of molecular markers for efficient use in the characterization of desirable genotypes and genetic diversity analysis^14^.

In this study, carnation mutants were characterized at the molecular level with an objective of releasing the newly developed mutants for commercial cultivation and later their utilization in breeding programmes. The aim of inducing mutations in the carnation was to develop high-yielding varieties with improved quality and novel colours to increase its diversity. Development of new cultivars by spontaneous mutation techniques occurs at low frequency, whereas induced mutation techniques create variability in flower crops at higher frequency^15^. To produce stable, non-chimeric mutants in carnation, *in vitro* propagation techniques can be used in mutation breeding programmes.

## Objectives

- To identify putative mutants in carnation with improved traits for commercial cultivation.
- To identify and confirm variation among mutants at the genetic level by characterization using ISSR marker**s**.

## Materials and methods

### Experimental location and plant material

The experiment was conducted in the Department of Fruit Breeding,Punjab Agricultural University, Ludhiana and the Plant Tissue Culture Laboratory of the Department of Floriculture and Landscaping of Dr. YS Parmar University of Horticulture and Forestry, Nauni, Solan, Himachal Pradesh .

**Fig. 1:**
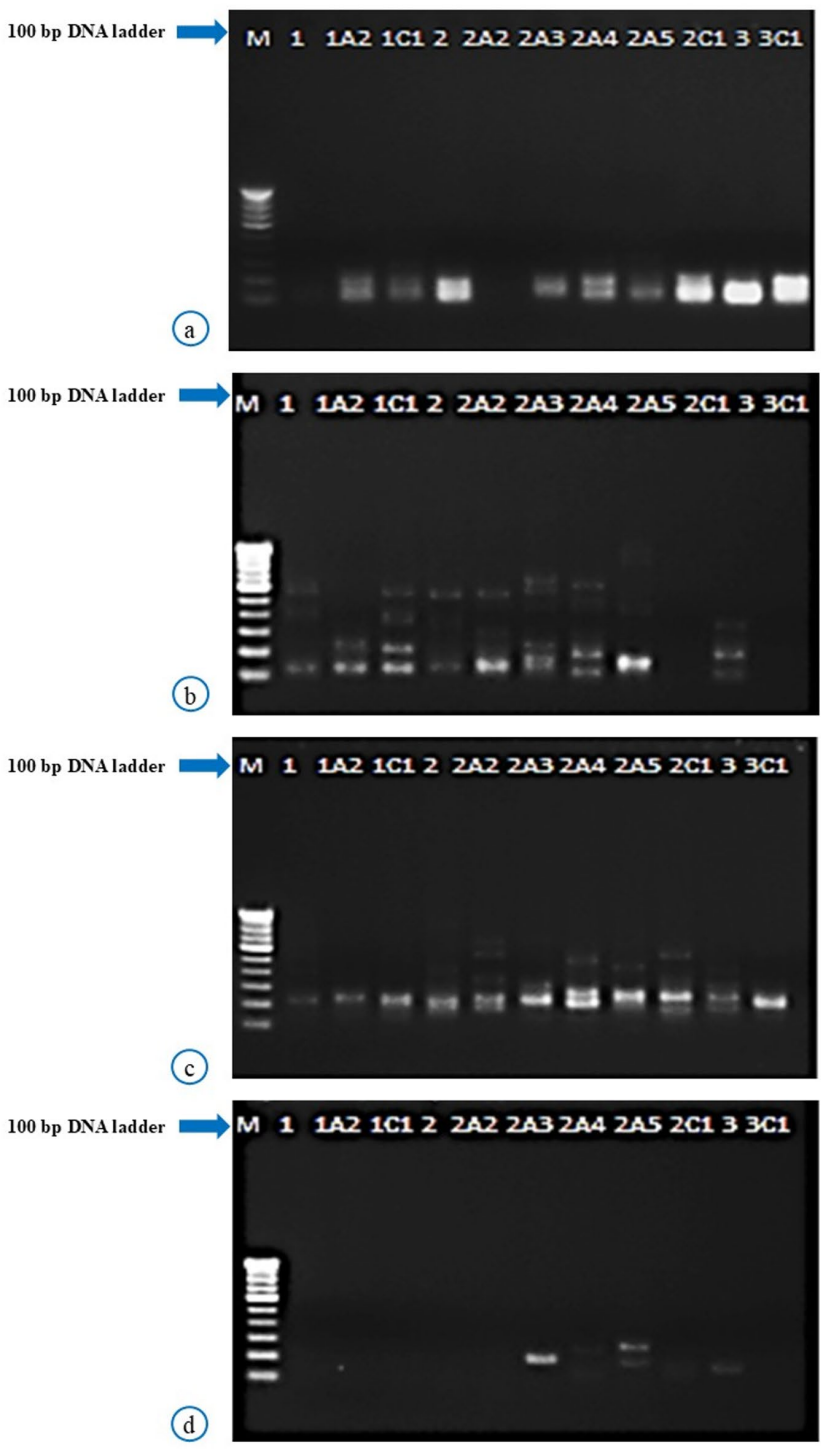
Banding pattern in carnation (*Dianthus caryophyllus* L.) parents and their mutants generated using different primers. M depicts the 100 bp DNA ladder utilized as a molecular size marker. **a**–**d** Illustrate the amplification profiles obtained with distinct ISSR primers: **a** ISSR-3, **b** ISSR-5, **c** ISSR-7 and **d** UBC-855. *Lane 1 indicates the parent ‘Tempo’; 1A2: Mutant ‘Tempo A_1_’; 1C1: Control. Lane 2 indicates the parent ‘Dark Rendez-Vous’; 2A2: Mutant ‘Rendez-Vous A_1_’; 2A3: Mutant ‘Rendez-Vous A_2_’; 2A4: Mutant ‘Rendez-Vous A_3_’, 2A5: Mutant ‘Rendez-Vous C_1_’ and 2C1: Mutant ‘Rendez-Vous C_2_’. Lane 3 indicates the parent ‘Madras’; 3C1: Mutant ‘Madras A_1_’.

In this experiment different cultivars of carnation viz., ‘Rendez-Vous’, ‘Tempo’and ‘Madras’ were used. The shoot cultures of these carnation cultivars were raised in the Plant Tissue Culture Laboratory of the Department.

### *In vitro* culturing and irradiation of cultured plants

**Fig. 2:**
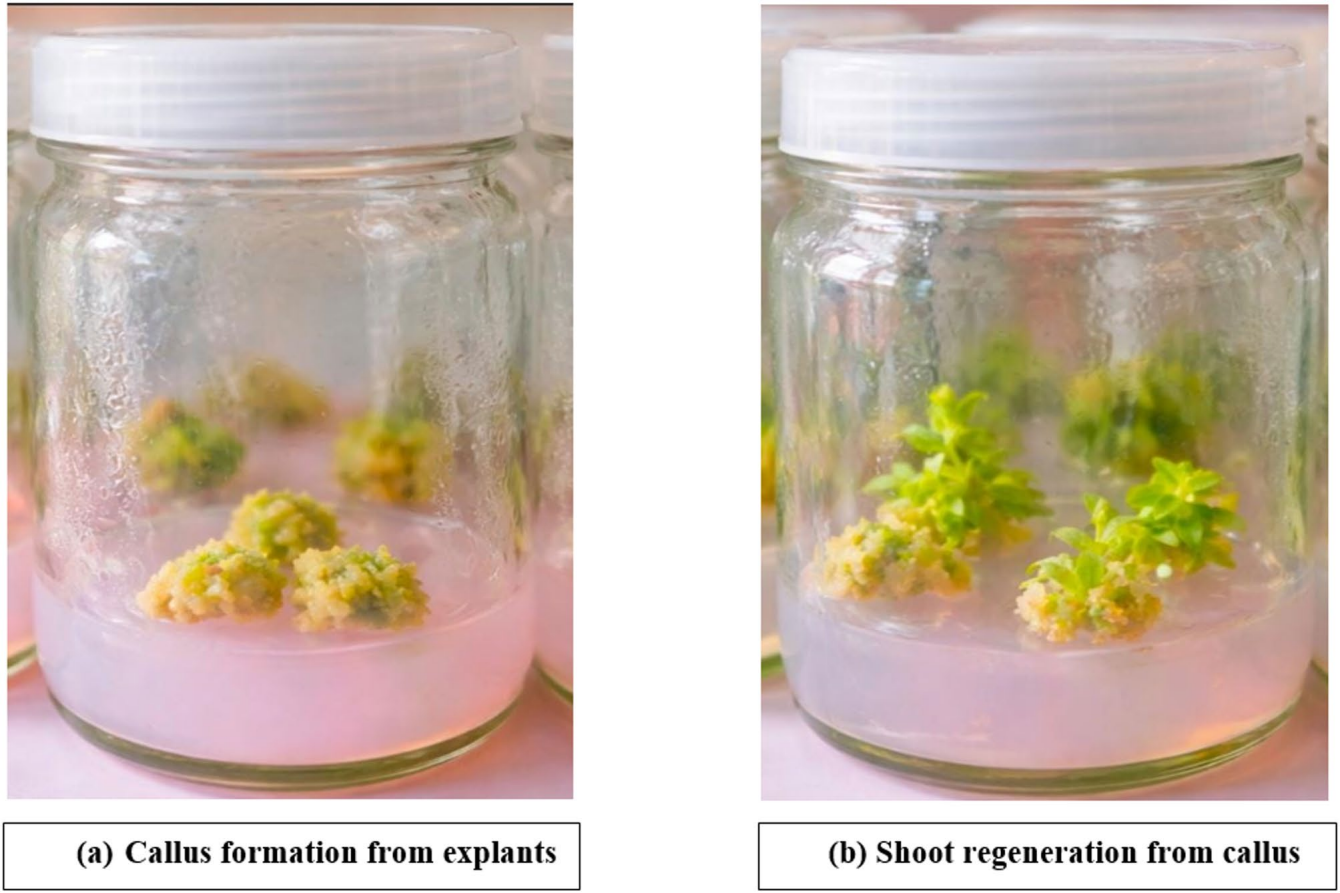
*In vitro* callus induction and shoot regeneration in carnation (*Dianthus caryophyllus* L.)

Firstly, shoots were cultured on MS medium supplemented with 2 mg/l BA (Fig. 2). These shoots were irradiated after one month of culturing at Gamma Chamber of the Department of Fruit Breeding, Punjab Agricultural University, Ludhiana. Shoot cultures were exposed to doses of gamma radiations like 30 Gy, 40 Gy and 50 Gy. After being exposed to irradiation the irradiated cultures were observed in a lab environment for around 20 days. Then, after one month, the percentage of mortality was calculated for the subcultures of the surviving cultures on multiplication media (2 mg/l BA + 1 mg/l GA3 + 0.1 mg/l NAA). The irradiated cultures were kept in laboratory conditions, and shoots were rooted in a medium containing MS + 2 mg/l NAA + 0.1% activated charcoal under *in vitro* conditions. After about a month of culturing, rooting was observed. Rooted shoots were then hardened in a medium consisting of cocopeat and sand (2:1, v/v). Then, after 20 days of hardening, plants were transferred to field conditions. To study mutation induction, the hardened plants were transplanted into the field after being acclimatised there under greenhouse conditions. Flowering was observed in mutants/variants and the data was recorded for different growth and flower characters are presented in Table 1. The flower colour was recorded using the Colour Charts of Royal Horticultural Society. Seven variants for flower colour were isolated from mutant generation (M_1_) obtained from different parents.

### Statistical data analysis for morphological traits

The data recorded were analyzed using SPSS statistics (Version 16.0, SPSS Inc., Chicago, IL, USA). The analysis of variance was performed for the data under a Randomized Block Design and mutants were compared using the critical difference at the 5% level of significance.

### Genomic DNA isolation

For further molecular research, different mutants along with their parents were used. Afterwards, healthy and fresh leaves from each plant at the four-pair leaf stage were collected and utilized for DNA isolation. The leaves were used to isolate the pure quality of DNA using the CTAB method given by Doyle and Doyle^16,17^. Chloroform-phenol treatment followed by suspension and precipitation using absolute alcohol, was used for further purifying the isolated DNA. The pellet was then dissolved in Tris EDTA (TE) buffer (10 mM Tris-HCl and 1mM EDTA, pH 8) and stored at 4 °C untill further use. A UV/VIS spectrophotometer (Perkin Elmer) and 0.8 per cent agarose gel stained with ethidium bromide were used, respectively, to analyze the quantity and quality of DNA.

### PCR amplification of genomic DNA

PCR (Polymerase Chain Reaction) amplification of genomic DNA was performed with ten ISSR primers (Inter Simple Sequence Repeats) as mentioned in the Table 1. With both marker systems, a reaction mixture of 20 µl was used for the experiment. This reaction mixture contained 1 x PCR buffer A (containing 1.5 mM MgCl_2_), 1 mM dNTPs, 10 picomoles of primer, 1 U of Taq DNA polymerase, 30 ng of DNA and autoclaved distilled water to make up the final volume of 20 µl. The thermal profile used for amplification with ISSR was: initial denaturation for 2 min at 94 °C, followed by 40 cycles of denaturation for 10 s at 94 °C, annealing according to the primer Tm, extension for 10 min at 72 °C, and a final extension for 10 min at 72 °C^18^.

**Table 1:** List of ISSR primers used in present study for DNA amplification.

S. No.	Primer code	Primer sequence (5′→3′)	GC Content* (%)	Tm** (°C)	Number of bands produced
1	ISSR-3	GAGAGAGAGAGAGAGAYC	52.8	55	2
2	ISSR-5	AGAGAGAGAGAGAGAGYC	52.8	55	5
3	ISSR-7	ACACACACACACACACYC	52.8	55	6
4	UBC-855	ACACACACACACACACCTT	47.4	55	2
5	Primer 1	CACACACACACACACAAGG	52.6	56	0
6	ISSR 2	CAGAGAGAGAGAGAGAYT	47.2	53	0
7	UBC840	GAGAGAGAGAGAGAGACTC	52.6	57	0
8	IISRS-3-E	ACACACACACACACACG	52.9	52	0
9	IISRS-3-G	GTGGTGGTGGTGGTG	66.7	52	0
10	IISRS-3-H	GACGACGACGACGAC	66.7	52	0

*GC Content: Guanine-Cytosine content, **Tm: Annealing temperature, ISSR primers were designed on the basis of microsatellite repeat motifs which are numerous across plant genome.

### Electrophoresis of amplified DNA

The amplified DNA was thoroughly mixed with 6x loading dye (0.25% bromophenol blue, 40% sucrose) and then electrophoresed in a 1.2% agarose gel for ISSRs in 1x TAE buffer (40 mM Tris-acetate, 1.0 mM EDTA)^19^. Ethidium bromide @ 0.5 µg ml^−1^ was incorporated into the gel. Then, for about three hours the gel was run at a constant voltage @ 5 V cm^−1^.

### Banding profile analysis

Bands amplified by different markers were compared for similarities and dissimilarities. Gel images of ISSR marker system were converted into binary matrices (Fig. 1a–d). Anderson et al.^20^, Smith et al.^21^ and Roy et al.^22^ described the method to determine polymorphic information content (PIC value) for each primer. The ability of each primer to distinguish between cultivars was measured using the resolving power (Rp) formula, Rp = PIb, where Ib denotes band informativeness. The effective multiplex ratio (EMR) was obtained using the formula EMR = nb, and marker index (MI) was calculated as a product of the PIC and the EMR^23^. For the calculation of genetic diversity, POPGENE version 1.31 was used, and parameters like Nei’s gene diversity (h) and Shannon information index (I) were determined. Gene flow (Nm) was calculated as Nm = 0.50 £ (1 − GST/GST). The genetic structure of the population was examined using a Bayesian clustering analysis method in STRUCTURE software version 2.3.4^24^. The DK method was used to determine the appropriate K-value and distinct clusters (K) was expected to be between 1 and 6, with 100,000 independent runs for each ‘K’. POPGENE version 1.31 was used to create a pairwise similarity index using Jaccard coefficients which was then subjected to UPGMA cluster analysis and a dendrogram was created^25^. The GenAlEx version 6.5 was used to identify the level of significant variation among and within populations, and the fixation index or F-statistics (FST) was also determined^26^.

### Data analysis

The bands of mutants were compared with the bands corresponding to the parents. Polymorphism between parents and mutants indicated the presence of variations, which further confirmed the presence of mutation at the genetic level.

## Results

### Induction of mutation and selection of genotypes

The analysis of variance for all the characters under study revealed significant differences among the mutants in comparison to the parents. The mean performance of ten genotypes of carnation has been described separately for different vegetative and flowering parameters. In case of cv. ‘Rendez-Vous’ three mutants were observed at gamma radiation of 30 Gy and two mutants were observed at a radiation of 50 Gy. While in the case of cvs. ‘Tempo’ and ‘Madras’, one mutant each was observed at gamma radiation of 30 Gy. 

In the leaves and flowers of mutants, the occurrence of morphological abnormalities was not observed. The main constraint in mutation breeding of flower crops is the formation of chimeras after mutagen treatment. It is possible to isolate chimeras if they occur in whole branch, although it becomes difficult to isolate them when a sector of the flower is mutated. In the present investigation, all the mutants were found stable in nature, having same colour of florets or petals, with no evidence of chimera formation.

### Morphological parameters of carnation mutants and parents

#### Vegetative parameters

Vegetative parameters i.e. days taken to flower bud formation, bud diameter, bud length, thickness of flower stem, stem length and plant height for ten genotypes with their respective parents were recorded, which are presented in Table 1.

Data presented in Table 2 about days taken to flower bud formation revealed that among different genotypes, ‘Rendez-Vous’ (127.27 days) took the minimum number of days for flower bud formation, which was found significant with ‘Rendez-Vous A_2_’ (127.40 days), ‘Madras’ (128.53 days), ‘Rendez-Vous A_3_’ (128.87 days), ‘Rendez-Vous C_2_’ (128.93 days) and ‘Rendez-Vous A_1_’ (130.80 days). On the other hand,  the maximum number of days taken for flower bud formation was recorded in the genotype ‘Tempo’ (135.93 days) followed by ‘Rendez-Vous C_1_’ (132.27 days). While comparing different mutants with their parents, ‘Rendez-Vous’ took fewer days for bud formation (127.27 days), followed by mutants ‘Rendez-Vous A_2_’ (127.40 days), ‘Rendez-Vous A_3_’ (128.87 days), ‘Rendez-Vous C_2_’ (128.93 days). Hence, these mutants can be considered for early flowering. On the other hand, ‘Rendez-Vous C_1_’ (132.27 days) took the maximum number of days followed by ‘Rendez-Vous A_1_’ (130.80 days) and can be considered for late flowering. The observed variations highlight the potential for staggered flowering.

**Table 2:** Effect of gamma irradiation on morphological (vegetative and flowering) characters of carnation (*Dianthus caryophyllus* L.) mutants.

Mutants	Parameters										
	γ- ray dose	Flower colour		Days taken to flower bud formation	Bud diameter (mm)	Bud length (mm)	Thickness of flower stem (mm)	Stem length (cm)	Plant height (cm)	Days to harvesting of cut stems (days)	Calyx splitting (%)
		Petal colour	Petal colour								
Rendez-Vous	Parent	Purple Violet Group 82D	Red Purple Group 72B	127.27	18.07	25.44	4.47	41.21	59.45	161.87	0.00
Rendez-Vous A_1_	30 Gy	Yellow Group 2D	Red Purple Group 59B	130.80	19.05	26.46	4.91	45.66	59.30	156.92	0.84
Rendez-Vous A_2_	30 Gy	Red Group 36D	Red Purple Group 71 A	127.40	18.81	25.58	4.89	55.90	67.70	157.44	0.84
Rendez-Vous A_3_	30 Gy	Violet Group 87 C	Violet Purple Group 71 C	128.87	19.45	28.43	5.10	56.71	66.05	143.67	0.00
Rendez-Vous C_1_	50 Gy	Red Purple Group 71B	–	132.27	19.89	27.22	4.97	46.00	63.49	157.07	0.25
Rendez-Vous C_2_	50 Gy	Red Purple Group 61B	–	128.93	19.39	27.86	5.40	45.55	65.94	165.40	0.00
Tempo	Parent	Red Group 36D	Red Purple Group 59B	135.93	17.84	24.63	4.89	48.65	61.08	158.95	0.00
Tempo A_1_	30 Gy	Yellow Group 11D	Red Purple Group 60D	132.13	20.20	27.34	5.60	49.34	59.18	164.23	0.00
Madras	Parent	Yellow Group 2D	Red Purple Group 60 A	128.53	18.89	25.48	4.67	43.40	59.98	171.43	0.00
Madras A_1_	30 Gy	Yellow Group 4D	Red Purple Group 60 A	131.13	20.20	26.08	4.79	44.67	62.55	166.09	0.00
CD_0.05_	–	–	–	3.67	0.35	0.32	0.38	1.18	1.30	1.08	0.42

CD_0.05_: Critical difference at the 5% significance level.

Data about bud diameter revealed that the maximum bud diameter among different genotypes was recorded in ‘Tempo A_1_’ and ‘Madras A_1_’ (20.20 mm), which was found significant with ‘Rendez-Vous C_1_’ (19.89 mm). Whereas, minimum bud diameter was recorded in ‘Tempo’ (17.84 mm) which was found significant with ‘Rendez-Vous’ (18.07 mm). Among different genotypes, the maximum bud length was recorded in ‘Rendez-Vous A_3_’ (28.43 mm). On the other hand, the minimum bud length was recorded in ‘Tempo’ (24.63 mm). Among different genotypes maximum thickness of flower stem was recorded in ‘Tempo A_1_’ (5.60 mm) which was found significant with ‘Rendez-Vous C_2_’ (5.40 mm). Whereas, minimum thickness of flower stem was shown by the genotype ‘Rendez-Vous’ (4.47 mm) followed by ‘Madras’ (4.67 mm) and ‘Madras A_1_’ (4.79 mm). Data related to stem length revealed that the maximum stem length was recorded in genotype ‘Rendez-Vous A_3_’ (56.71 cm), which was found significant with ‘Rendez-Vous A_2_’ (55.90 cm). Whereas, the genotype ‘Rendez-Vous’ showed the minimum stem length (41.21 cm).

Among different genotypes, the maximum plant height was recorded in ‘Rendez-Vous A_2_’ (67.70 cm).Whereas, the minimum plant height was recorded in ‘Tempo A_1_’ (59.18 cm), which showed significant results with genotype ‘Rendez-Vous A_1_’ (59.30 cm), ‘Rendez-Vous’ (59.45 cm) and ‘Madras’ (59.98 cm). Among different mutants of ‘Rendez-Vous’ (59.45 cm) the minimum height was recorded in ‘Rendez-Vous A_1_’ (59.30 cm) and maximum height was recorded in ‘Rendez-Vous A_2_’ (67.70 cm), followed by ‘Rendez-Vous A_3_’ (66.05 cm), ‘Rendez-Vous C_2_’ (65.94 cm) and ‘Rendez-Vous C_1_’ (63.49 cm). Variations recorded suggest that these mutants could be targeted for specific commercial attributes, such as plants with maximum height for high-grade cut flowers.

#### Flowering parameters

**Fig. 3:**
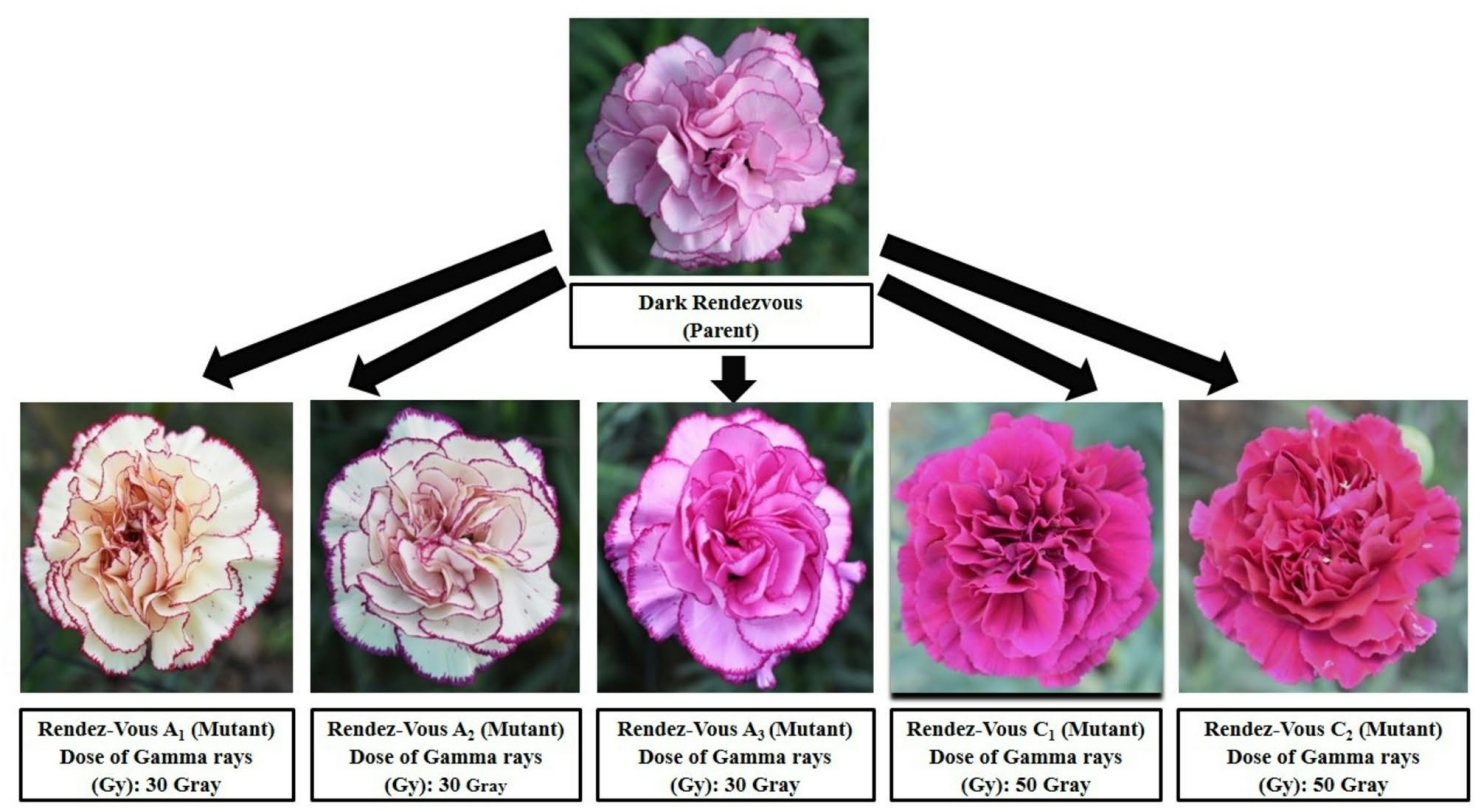
Variation in gamma-irradiated mutants from the parent ‘Dark Rendezvous’.

**Fig. 4:**
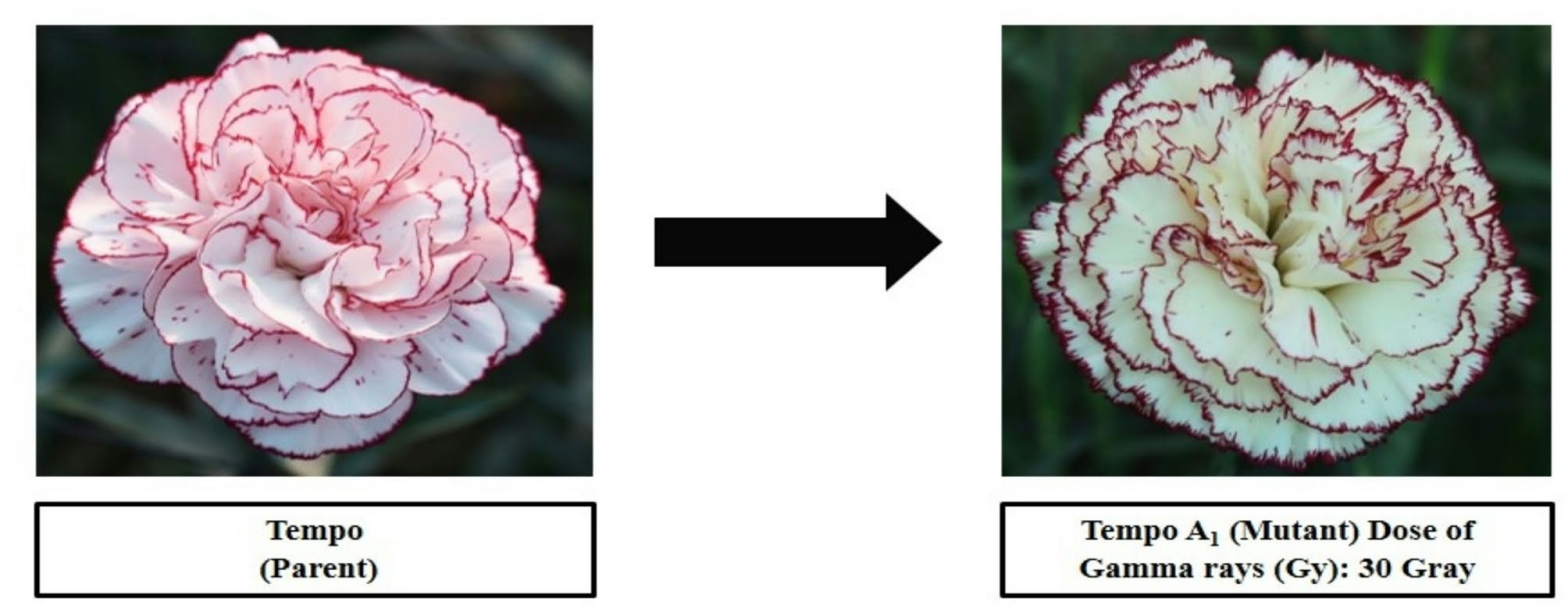
Variation in gamma-irradiated mutant from the parent ‘Tempo’.

**Fig. 5:**
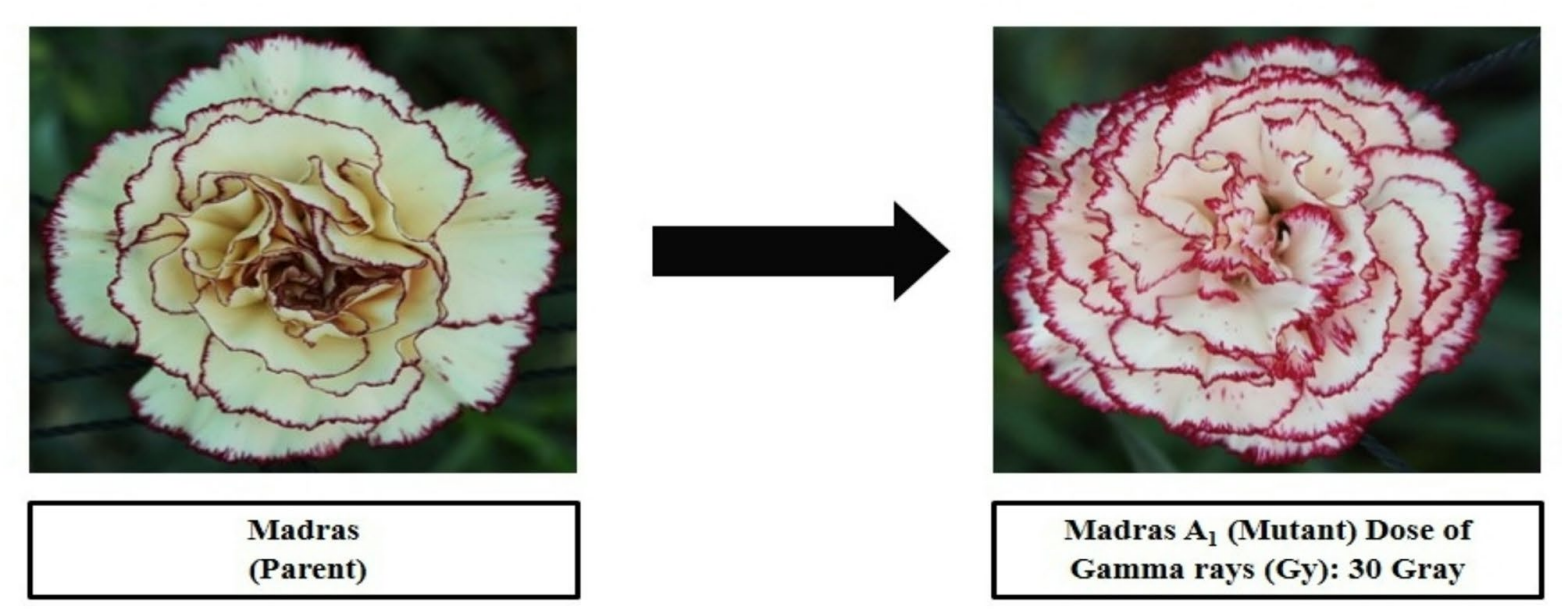
Variation in gamma-irradiated mutant from the parent ‘Madras’.

Comparing these mutants to their parents, it is clear that they exhibit significantly different colours and petal margin patterns. Data presented in Table 2 show that at 30 Gy gamma radiation three mutants were recorded in cv. ‘Rendez-Vous’ which showed different colours of petals and margins as compared to parent. The primary petal colour of cv. ‘Rendez-Vous’ was Purple Violet Group 82D and the margin colour was Red Purple Group 72B as shown in Fig. 3. The primary colour of ‘Rendez-Vous A_1_’ and ‘Rendez-Vous A_2_’ was observed to be Yellow Group 2D and Red Group 36D respectively, and secondary petal colour was Red Purple Group 59B and Red Purple Group71A. Whereas, ‘Rendez-Vous A_3_’ recorded a primary colour of Violet Group 87 C with a secondary colour of Violet Purple Group 71 C. Primary petal colour in the case of ‘Rendez-Vous C_1_’ and ‘Rendez-Vous C_2_’from cv. ‘Rendez-Vous’ was observed to be Red Purple Group 71B and Red Purple Group 61 A. The whole petals had the same colour in ‘Rendez-Vous C_1_’and ‘Rendez-Vous C_2_’. In the case of cv. ‘Tempo’ the primary colour of petals and margin was recorded as Red Group 36D and Red Purple Group 59B respectively. Primary petal colour in mutant ‘Tempo A_1_’ was recorded to be Yellow Group 11D and the secondary petal colour was Red Purple Group 60D as shown in Fig. 4. Whereas, the primary petal colour of cv. ‘Madras’ and its mutant ‘Madras A_1_’ was Yellow Group 2D and Yellow Group 4D respectively. The secondary margin colour of cv. ‘Madras’ and ‘Madras A_1_’ was Red Purple Group 60A as shown in Fig. 5. Variations in flower colour after irradiation of shoots might have resulted due to deletions in chromosome structure or chromosomal aberrations. These results demonstrate that mutation breeding can produce distinct genotypes, which hold potential for commercial cultivation.

Flowering parameters i.e. days to the harvesting of cut stems, flower size, number of petals per flower, petal characters (width and height), number of flowers per plant and per meter square, number of marketable flowers per plant and per meter square, duration of flowering, vase life and calyx splitting for seven mutants with their respective parents were recorded which are presented in Tables 2 and 3. Among different mutants, the minimum number of days taken for harvesting of cut stems was recorded in the ‘Rendez-Vous A_3_’ (143.67 days) and the maximum number of days was taken by ‘Madras’ (171.43 days). Further, while comparing different mutants, maximum flower size was recorded in the mutant ‘Rendez-Vous C_2_’ (8.40 cm). On the other hand, the minimum flower size was recorded in the ‘Madras A_1_’ (7.10 cm), which was found significant with ‘Tempo A_1_’ (7.20 cm). Among different mutants of ‘Rendez-Vous’ (7.57 cm), the maximum flower size was recorded in ‘Rendez-Vous C_2_’ (8.40 cm), ‘Rendez-Vous A_1_’ (8.35 cm), ‘Rendez-Vous A_3_’ (8.15 cm), ‘Rendez-Vous A_2_’ (7.60 cm) and ‘Rendez-Vous C_1_’ (7.60 cm). Among different mutants, a higher number of petals per flower was recorded in ‘Rendez-Vous C_2_’ (78.00), while a lower number of petals per flower was found in ‘Tempo’ and ‘Tempo A_1_’ (44.00).

**Table 3:** Effect of gamma irradiation on morphological (flowering) characters of carnation (*Dianthus caryophyllus* L.) mutants.

Mutants	Parameters										
	Fragrance	Flower size (cm)	Number of petals per flower	Petal characters (cm)		Number of flowers per plant	Number of flowers per meter square	Number of marketable flowers per plant	Number of marketable flowers per meter square	Duration of flowering (days)	Vase life (days)
				Width	Height						
Rendez-Vous	Present	7.57	65.00	3.72	5.61	4.67	116.67	4.20	105.00	48.33	10.17
Rendez-Vous A_1_	Present	8.35	70.00	4.00	5.97	5.00	125.00	4.33	108.25	57.33	12.24
Rendez-Vous A_2_	Present	7.60	74.00	3.80	5.83	4.73	118.33	4.13	103.25	52.67	12.10
Rendez-Vous A_3_	Present	8.15	59.00	4.27	6.13	4.33	108.33	4.00	100.00	65.67	11.95
Rendez-Vous C_1_	Absent	7.60	62.00	4.33	5.77	4.73	118.33	4.00	100.00	62.67	14.79
Rendez-Vous C_2_	Present	8.40	78.00	4.47	6.07	4.47	111.67	4.33	108.25	54.67	15.27
Tempo	Present	8.00	44.00	3.63	5.53	4.13	103.33	3.66	91.67	63.33	12.60
Tempo A_1_	Present	7.20	44.00	3.47	5.33	4.20	105.00	4.00	100.00	67.67	14.20
Madras	Present	7.60	50.00	4.27	5.27	3.66	91.67	3.33	83.33	52.67	11.33
Madras A_1_	Present	7.10	53.00	3.13	5.33	4.67	116.67	4.20	105.00	65.20	14.70
CD_0.05_	–	0.23	1.90	0.21	0.26	0.42	9.44	0.43	9.27	3.20	0.54

CD_0.05_: Critical difference at the 5% significance level.

**Fig. 6:**
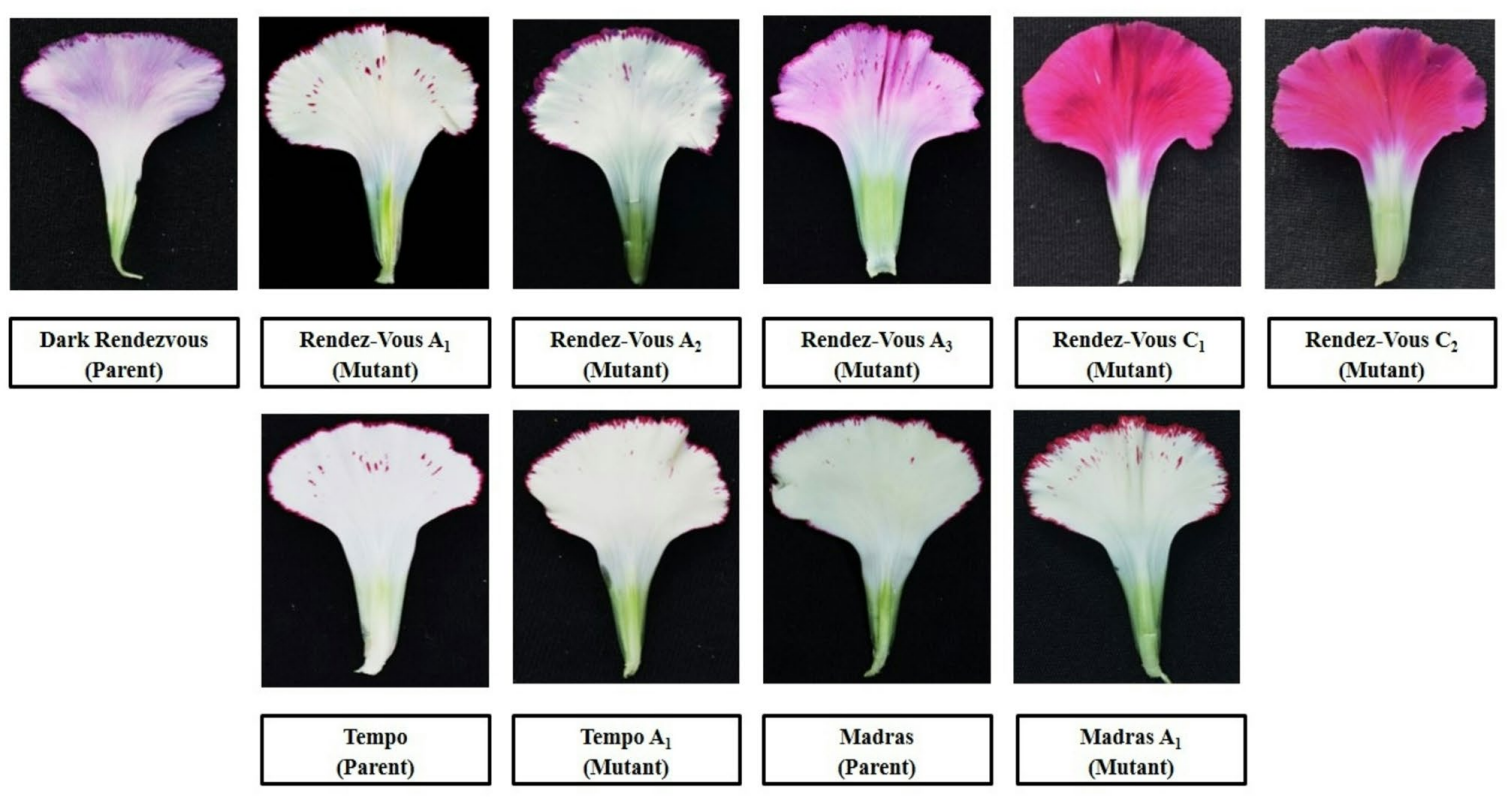
Variation in primary and secondary petal colour of different mutants and their parents.

Among different mutants, petal colour (primary and secondary) varied as shown in Fig. 6. Petal width was recorded as maximum in ‘Rendez-Vous C_2_’ (4.47 cm), which was found significant with ‘Rendez-Vous A_3_’ and ‘Madras’ (4.27 cm). Whereas, the minimum petal width was recorded in ‘Madras A_1_’ (3.13 cm).  The maximum petal height among different mutants was recorded in ‘Rendez-Vous A_3_’ (6.13 cm). On the other hand, the minimum petal height was recorded in ‘Madras’ (5.27 cm) followed by ‘Tempo A_1_’, ‘Madras A_1_ (5.33 cm) and ‘Tempo’ (5.53 cm). The number of flowers per plant and per meter square among different mutants was found to be maximum in ‘Rendez-Vous A_1_’ (5.00, 125.00), respectively. It was found to be at par with ‘Rendez-Vous A_2_’ (4.73, 118.33), ‘Rendez-Vous C_1_’ (4.73, 118.33), ‘Rendez-Vous’ (4.67, 116.67) and ‘Madras A_1_’ (4.67, 116.67), respectively. Whereas, the minimum number of flowers per plant and per meter square were found in ‘Madras’ (3.66, 91.66), respectively. Among different mutants of ‘Rendez-Vous’ (4.67, 116.67), the number of flowers per plant and per meter square was found to be higher in ‘Rendez-Vous A_1_’ (5.00, 125.00), ‘Rendez-Vous A_2_’ (4.73, 118.33) and ‘Rendez-Vous C_1_’ (4.73, 118.33), respectively, when compared to the parent. ‘Madras A_1_’ recorded a higher number of flowers per plant and per meter square (4.67, 116.67) as compared to the parent ‘Madras’ (3.66, 91.67), respectively.

The number of marketable flowers per plant and per meter square was found to be maximum in ‘Rendez-Vous A_1_’ (4.33, 108.25), respectively; on the other hand, minimum number of marketable flowers per plant and per meter square were found in ‘Madras’ (3.33, 83.33), respectively. Duration of flowering was recorded as maximum in ‘Tempo A_1_’ (67.67 days) and was found to be at par with ‘Rendez-Vous A_3_’ (65.67 days) and ‘Madras A_1_’ (65.20 days). Shorter duration of flowering was recorded in ‘Rendez-Vous’ (48.33 days). All the mutants of ‘Rendez-Vous’ recorded a longer duration of flowering compared to the parent i.e. ‘Rendez-Vous A_3_’ (65.67 days) followed by ‘Rendez-Vous C_1_’ (62.67 days), ‘Rendez-Vous A_1_’ (57.33 days), ‘Rendez-Vous C_2_’ (54.67 days) and ‘Rendez-Vous A_2_’ (52.67 days). The mutants of the parents ‘Tempo’ (63.33 days) and ‘Madras’ (52.67 days) also recorded a longer duration of flowering i.e. ‘Tempo A_1_’ (67.67 days) and ‘Madras A_1_’ (65.20 days), respectively. Vase life was recorded as maximum in ‘Rendez-Vous C_1_’ (14.79 days) and was found to be at par with ‘Madras A_1_’ (14.70 days). The minimum vase life was recorded in the ‘Rendez-Vous’ (10.17 days). All the mutants of ‘Rendez-Vous’ (10.17 days) recorded a longer vase life compared to the parent i.e. ‘Rendez-Vous C_2_’ (15.27 days), ‘Rendez-Vous C_1_’ (14.79 days), ‘Rendez-Vous A_1_’ (12.24 days), ‘Rendez-Vous A_2_’ (12.10 days) and ‘Rendez-Vous A_3_’ (11.95 days). Mutants of parent ‘Tempo’ (12.60 days) and ‘Madras’ (11.33 days) also recorded a longer vase life i.e. ‘Tempo A_1_’ (14.20 days) and ‘Madras A_1_’ (14.70 days), respectively. The variations recorded indicate that mutants surpass their parents in significant commercial parameters, contributing their use in targeted breeding.

### ISSR based diversity analysis

#### ISSR studies and genetic polymorphism

Carnation mutants along with parents were characterized using ten ISSR primers and DNA polymorphism was observed with four primers. A total of fifteen polymorphic bands were produced by four primers. No monomorphic band was found with any primer which resulted in 100% polymorphism as shown in Fig. 1. Variations in the plant material used can be seen through genetic polymorphism. Hence, the conclusion drawn from this study was that putative mutants truly varied at the DNA level.

#### Analysis of molecular variance (AMOVA)

AMOVA performed using ISSR marker demonstrated that 85% of the variation was recorded within individuals and 15% among the populations, respectively. This pattern corresponds with the morphological variation recorded, indicating that variations such as stem length, flower size and duration of flowering are partially associated with genetic variation revealed through ISSR markers.

## Discussion

Mutation breeding when combined with tissue culture, is an effective approach for introducing desirable characteristics and developing novel varieties of commercial utility in vegetatively propagated crops. Hence, this technique was used to develop variation in flower colour along with other desirable characters. According to Mehta et al.^27^ the plants regenerated from selected calli exhibited tolerance against diseases.

Sahavacharin and Suriyasak^28^ irradiated shoot tips (1.5-2.0 cm long) of cv. ‘White Sim’, which were cultured on MS medium with successive increases in radiation dose, and observed that after 12 weeks of treatment, survival and growth of shoots declined significantly. The plants irradiated at doses of 1 to 3 Kr recorded variations, having pink streaks on white petals as compared to the control. Simared et al.^7^ used gamma rays to treat the *in vitro *raised callus from petals of cv. ‘Niky’. On the fourth day of culture, when explants were exposed to 50 Gy dose, variants of white flowers with pink dots, streaks and borders were produced in cv. ‘Niky’ which may have arisen due to mutation of a recessive gene encoding anthocyanin. The observations recorded in the present investigation showed significant variation for the different vegetative parameters and flowering parameters. These variations might be due to physiological changes and the genetic makeup of the mutants and parents. Similar variations were recorded by Chauhan et al.^29^, Jose et al.^30^, Maurya et al.^31^, Sharma et al.^32^ for parameters i.e. number of days taken to flower bud formation, bud diameter, stem thickness and plant height in carnation.

Significant variations in flowering parameters i.e. flower size, duration of flowering and vase life could be attributed to prevailing climatic conditions and differences in genetic composition of the mutants. Wide variation for flowering parameters in carnation was also noted by Sarkar and Sharma^33^, Chauhan et al.^29^, Maurya et al.^31^ and Sharma et al.^32^. Similar variations in the number of flowers per plant and marketable flowers per plant could be due to different genetic makeup of the mutants and prevailing climatic conditions. Comparable results were observed by Taghizadeh and Khadivi^34^ and Somaya et al.^35^ in carnation. Calyx splitting might have occurred due to lower temperature (< 10 °C) during the flower bud development, fluctuations in temperature and different genetic composition of the mutants. Similar variations were observed by Gharge et al.^36^ and Taghizadeh and Khadivi^34^ in carnation.

The conventional approach for analyzing genetic variations among plant species is based predominantly on morphological characteristics, but it has a major drawback that it is highly dependent on environmental conditions, which influence the reliability of results. To overcome this constraint, quick and precise molecular marker-based approaches have been developed using molecular biology techniques (Tejaswini et al.^37^, Wang et al.^38^, Sharma et al.^39^). Molecular markers are prominent tools in the field of molecular genetics due to their stability, low cost and ease of use for a wide range of applications^40^. ISSR, a PCR-based marker, is one of the most widely used markers for analysing genotypic changes in a variety of crop species^35,41,42^. These markers can help with parent selection and cultivar identification in breeding programmes^39^.

ISSR markers were also used for diversity analysis, which identified differences between carnation genotypes and mutants. ISSR analysis was used by Zietkiewicz et al.^43^ to establish relationships and variability in plant species, and ISSR markers have been documented as dominant markers in many studies for genomic characterization and have also been used for molecular characterization of carnation genotypes, using ISSR markers and obtaining similar results^2,44,45^. A similar investigation found 90.64% polymorphism in Jasmine sp. for genetic diversity^46^. Assessment of genetic diversity by ISSR markers has also been done in gladiolus by Chaudhary et al.^47^ and Jadhav et al.^48^. ISSR markers were found to be more efficient in analyzing genetic diversity in chrysanthemum by Baliyan et al.^49^ and Mekapogu et al.^50^. Similar research has been conducted in gerbera^51,52^, in spring orchid^53,54^ and in rose^55–57^. The improvement of a floricultural crop such as carnation is directly dependent on the characterization of genetically different genotypes. As a result, the genetic differences observed in the current study could be valuable in breeding programmes.

## Conclusion

The current study revealed that genetic diversity was found among different carnation mutants and their parents both at phenotypic as well as genotypic levels. Among the different mutants, ‘Rendez-Vous A_2_’, ‘Rendez-Vous A_3_’, ‘Rendez-Vous C_1_’ ‘Tempo A_1_’ and ‘Madras A_1_’ performed better for stem length, plant height, thickness of flower stem, flower size, vase life, yield and duration of flowering. At the genotypic level, ISSR markers proved to be useful in determining the genetic relationship among mutants and their parents. The results of this study illustrate that ISSR markers proved to be effective, producing distinctive bands that facilitated the identification of cultivars in carnation breeding programmes. These markers have been used to assess genetic differentiation and determine the evolutionary relationship between mutants and their parents. Because of their specificity and dominance, the ISSR markers utilized in this study were identified to be more reliable and effective, as they enabled the production of reproducible banding patterns.

## Data Availability

All data generated or analysed during this study are included in this article.
